# “Targeted” Chemotherapy for Esophageal Cancer

**DOI:** 10.3389/fonc.2017.00063

**Published:** 2017-04-03

**Authors:** Joe Abdo, Devendra K. Agrawal, Sumeet K. Mittal

**Affiliations:** ^1^Department of Clinical and Translational Science, Creighton University School of Medicine, Omaha, NE, USA; ^2^Norton Thoracic Institute, St. Joseph’s Hospital and Medical Center, Dignity Health, Phoenix, AZ, USA

**Keywords:** esophageal cancer, molecular diagnostics, targeted chemotherapy, targeted therapy, immunotherapy, precision medicine

Due to its increasing incidence and low survival rate, esophageal cancer (EC) is now considered, even by non-medical professionals, as an extremely difficult cancer to beat akin to well-known terminal indications such as pancreatic and lung cancers. The average 5-year survival of EC is less than 18% and currently has the fastest rising rate of incidence of all cancers diagnosed in the United States (1). A number of cancer types have had double-digit improvements in survival statistics in the last 30 years; however, EC has not experienced a jump in the positive direction in regards to improved outcomes even as the number of therapeutic options has grown (2). Having analyzed patient management strategies of hundreds of EC patients, it is apparent that survival rates are unlikely to improve significantly until there is an availability of new FDA-approved anticancer and immunotherapy drugs. Currently, most EC patients are prescribed a combination of platinums, taxanes, anthracyclines, or pyrimidine analogs. Targeted therapies [anti-receptor tyrosine-protein kinase erbB-2 (HER2) and anti-epidermal growth factor receptor (EGFR)] are sometimes used in concert with chemoradiation. Immunotherapy clinical trials for EC are in their infancy, especially in the first-line setting. The standard of care for patients with stages II–III EC usually consists of chemotherapy, or chemoradiation either in the neoadjuvant or adjuvant setting of esophagectomy (removal of the esophagus via surgical intervention). As of now, no grouping of chemotherapeutic agents has been clearly demonstrated to be the most effective combination therapy in the EC patient population. Thus, on top of dealing with the adverse effects of an extensive surgery, patients are subjected to sporadically ineffective chemotherapy regimens. This expensive and rigorous perioperative therapy protocol typically yields a short survival benefit over surgery alone (3, 4).

While we await a novel arsenal of therapeutic options in EC to be developed, what can surgeons, oncologists, scientists, and pathologists do to optimize first-line therapy to potentially improve survival rates? A straight-forward solution would involve research conducted in the recent past, current therapeutic options, and a more robust utilization of molecular diagnostics. Traditionally, proteomic expression assays are used in the targeted-therapeutic setting. For example, HER2 is a receptor associated with cancer progression and is expressed in approximately one of four gastroesophageal tumors (5, 6). Blocking HER2 with a drug such as trastuzumab would typically yield increased therapeutic efficacy and tumor regression (7). If a patient is prescribed trastuzumab and the tumor lacks HER2, the patient’s tumor is not likely to regress, leaving them only with toxic side effects such as cardiotoxicity (8). Thus, knowing whether this cellular surface protein is overexpressed in EC tumors is pertinent, which is why the expression status of HER2 is routinely ordered for this indication. This construct is also applicable with EGFR-targeted therapies. Even though the use of EGFR inhibitors is considered “off-label” in EC, knowing whether or not the tumor expresses EGFR is crucial when prescribing tyrosine kinase inhibitors (9). Clinical trials testing novel immunotherapies are incorporating proteomic expression of a patient’s tumors before entering the trial, as programmed death-ligand 1 (PD-L1) expression has been strongly associated with increased benefit when using drugs targeting PD-L1 (expressed on the tumor) and even PD-1 (expressed on the T-cell) (10, 11). It is imperative to affirm that the target being shot at is actually there. In recent years, researchers and clinicians have discovered proteomic biomarkers that have been linked to increased or decreased benefit when using certain classes of chemotherapy.

Of the 13 FDA-approved drugs for EC, 12 have affiliated biomarkers that are associated with either improved or decreased benefit in solid tumor indications (Figure 1). Thymidylate synthase (TS), if expressed in a tumor, is linked to decreased benefit when using the antimetabolite drugs capecitabine and fluorouracil (12). Excision repair cross complementation group 1 (ERCC1) is deemed a resistance marker to platinum-based therapies such as cisplatin, carboplatin, and oxaliplatin, which will have decreased benefit if expressed in a solid tumor (13–15). Topoisomerase 2*α* (TOPO2A) is an enzyme used in DNA repair and, if expressed, yields an increased benefit when using anthracyclines such as epirubicin and etoposide, drugs that target DNA repair mechanisms (16). Type I topoisomerase (TOPOI) is another protein involved in DNA annealing, and if expressed in cancer, is associated with increased benefit when using topoisomerase inhibitors such as irinotecan, which is often prescribed in EC (17, 18). Tubulin beta-3 (TUBB3) is another resistance marker that, when expressed, is linked with decreased benefit when using taxanes such as paclitaxel and docetaxel (19, 20). Reduced folate carrier (RFC), when expressed, is associated with improved benefit while using leucovorin, a drug that is used to a lesser extent in this indication but is still FDA approved for EC (21). Finally, as previously mentioned, HER2 is a companion biomarker to anti-HER2 drug trastuzumab, which is FDA approved in the first-line setting in EC. When HER2 is overexpressed, trastuzumab is more efficacious in halting tumor progression (22). Thus, 92% of the drugs that are FDA approved in EC have a concomitant biomarker that is associated with either increased or decreased benefit. The only drug in the cadre of EC therapy that lacks a proven diagnostic biomarker today is mitomycin.

**Figure 1:**
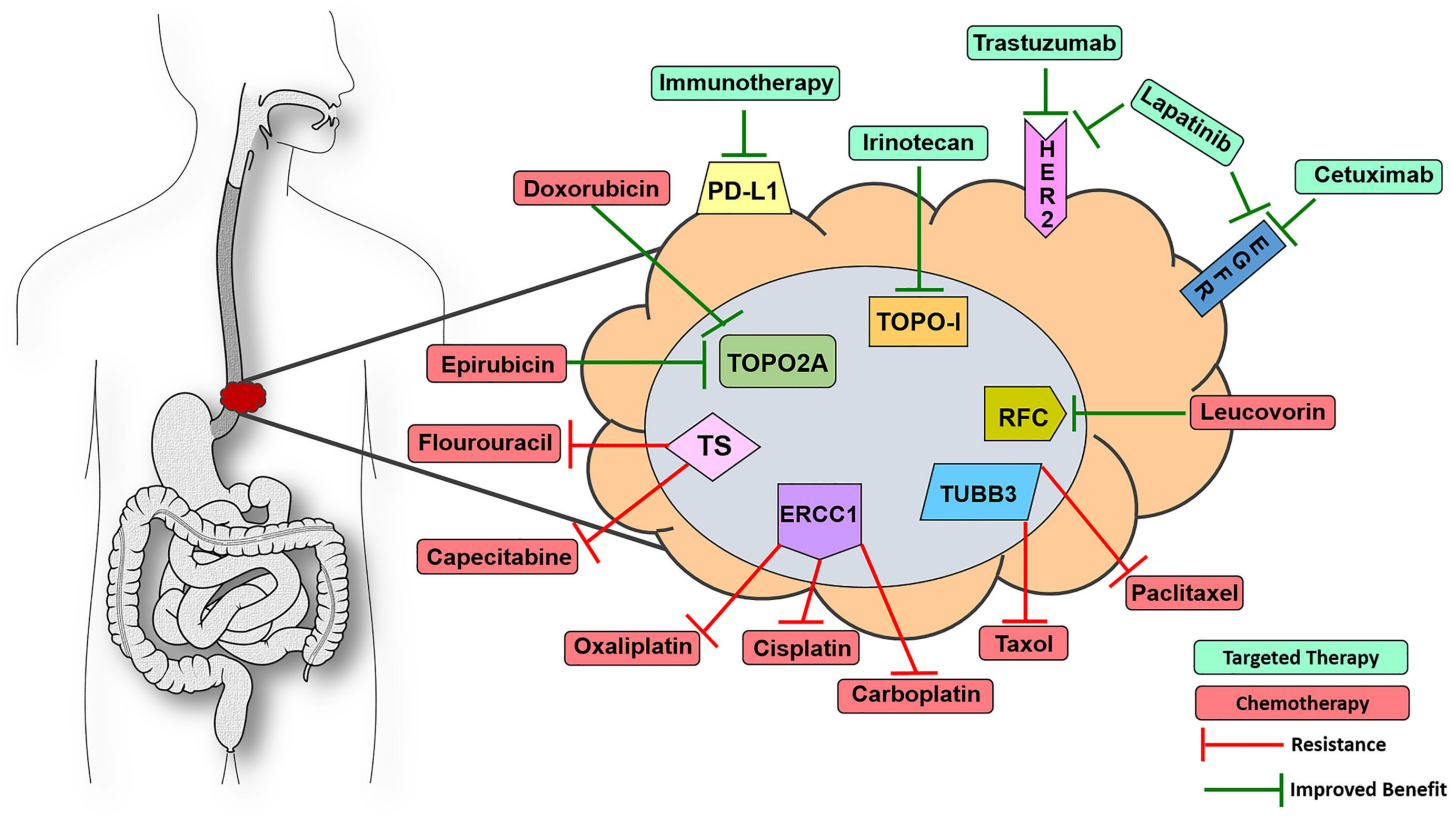
**Visualize esophageal cancer therapeutic targets with 9-marker panel**. Proteomic biomarkers affiliated with improved response to targeted therapy (green) or chemotherapy (red) drugs as well as resistance to various chemotherapeutic agents that are FDA approved for esophageal cancer (EC). Knowing the expression status of this 9-marker panel could assist oncologists in optimizing first-line patient management strategies for EC patients. Abbreviations: ERCC1, excision repair cross complementation group 1; TUBB3, tubulin beta-3; TOPO2A, topoisomerase 2α; TOPOI, type I topoisomerase; HER2, receptor tyrosine-protein kinase erbB-2; EGFR, epidermal growth factor receptor could assist; PD-L1, programmed death-ligand 1; RFC, reduced folate carrier; TS, thymidylate synthase.

In many cancers, there is an issue with the amount of tissue available for histological scrutiny before chemotherapy has been administered. However, a large percentage of EC patients undergo complete resection of the esophagus before adjuvant chemotherapy, which provides a large, unadulterated tumor with reliable protein expression because it remains unaffected by chemotherapy. These tumor samples contain ample amounts of tissue that could be used for outsourced diagnostics via private biotech companies or even in-house pathology-based assays. In this instance, oncologists and pathologists would order a 9-marker panel of proteomic expression assays covering virtually every drug in the EC therapy cache as an auxiliary to assembling first-line therapy, thereby improving the probability of prescribing an efficacious regimen (Figure 1). It would be more difficult to reliably analyze the tumors of patients who are treated with neoadjuvant chemotherapy before resection, as the only source of tissue for these patients is accessed via endoscopic pinch biopsies which are usually taken before chemo is administered. There are other pathology-based services that are required from this tissue, none of which are proteomic diagnostics for augmenting first-line therapy. However, if an oncologist was inclined to prescribe a combination of cisplatin + fluorouracil, it would be prudent to stain two formalin-fixed paraffin embedded sections for TS and ERCC1 to observe if the tumor possesses resistance markers for these drugs. Protein detection of two markers could potentially be determined by immunohistochemistry (IHC) of pinch biopsies only a few micrometers thick and would not necessitate using many large sections of tumor to detect the whole 9-marker panel. One of the side effects of cisplatin is that it can induce ototoxicity, rendering some patients completely deaf (23). Proteomics may be a useful strategy in avoiding unnecessary adverse events in the case of patients having a limited response to chemotherapy due to resistance elements in the tumor. Physicians assume the risk of low response rates and decreased efficacy by prescribing these chemotherapies in a relatively blind manner. There is little to lose by treating these patients with enhanced precision medicine because effective management of EC patients is one of the most difficult battles in the field of oncology.

Immunotherapy has tremendous potential to improve EC patient care based upon its impressive performance in other solid tumor types (24). Immediate screening for PD-L1 postesophagectomy would be most helpful in determining whether or not patients are ideal candidates for immunotherapy trials. PD-L1 is expressed in 40% of gastroesophageal cancers, so it cannot be presumed that all EC patients would be suitable candidates for PD-L1-targeted immunotherapy (25). However, placing PD-L1-positive patients in an immunotherapy clinical trial could potentially improve survival rates while simultaneously advancing this field of study.

The 9-marker panel proposed here (Figure 1) has not been specifically analyzed in a tumor registry or clinical trial, so whether or not this method would translate to improved patient outcomes is still unknown. However, commercial diagnostic companies are beginning to publish the results of test registries in which patient management strategies derived from their diagnostic platforms have translated into improved outcomes. For example, the *New England Journal of Medicine* published a study in 2015 demonstrating how a 21-gene expression test identified breast cancer patients who would receive no benefit from additional chemotherapy. Clinical use of this assay lowered the rate of recurrence as well as the number of adverse events in this population of breast cancer patients by treating them with endocrine therapy alone (26). Prognostic information derived from molecular diagnostics was demonstrated in a 2016 study, where investigators quantified HER2 in breast cancer samples with a commercial mass spectrometry assay as opposed to the +1, +2, and +3 quantification via IHC. Receptor tyrosine-protein kinase erbB-2 expression levels greater than 2,200 amol/μg was associated with significant extended disease-free survival and overall survival in adjuvant cases as well as longer overall survival in metastatic cases when treated with HER2-targeted therapy (27). So, quantitative proteomics also has the potential to predict the level of response to affiliated therapies.

Weighing the costs and expected benefits of molecular diagnostics is an unfortunate reality that must be considered when determining its feasibility when configuring first-line therapy strategies. Most oncology-based platforms can be extremely expensive, with tests ranging from $2,500–$34,000. Many payers are reluctant to offer even fractional reimbursement for diagnostic assays when there is no enough evidence of significant improvement in patient outcomes associated with the results of their tests. Oftentimes insurance will not offer any reimbursement to biotech companies for running their commercial assay in the clinical setting. However, most companies have financial assistance programs to more readily deliver results of their platform to oncologists and their patients. An alternate strategy would be the utilization of medical center pathology departments, where they should be able to perform IHC on this 9-marker panel using reimbursable ICD-9 codes at around $50–$200 per IHC stain. This would be an economical and expedited way to have information on the nine proteins that are relevant to EC therapy for a fraction of the price of an outsourced test.

To summarize, a diagnosis of EC is an uphill battle, no matter the stage the patient presents on day one. If a patient is fit for perioperative chemotherapy, screening their tumor for HER2, EGFR, PD-L1, ERCC1, TUBB3, TS, RCF, TOPOI, and TOPO2A would be a straightforward way to inform the oncologist which proteins in the patient’s tumors are prime targets and which proteins could potentially act as resistance molecules for EC drug options (Figure 1). RNAseq and genomic sequencing are often utilized by molecular diagnostic companies; however, the data in those assays not only provide extraneous information with little clinical utility to-date but also act as a surrogate for what is actually occurring in the proteome at the day of diagnosis. Therefore, screening EC tumors with the proposed 9-marker panel would inform oncologists of the expression status of the proteins associated with clinical utility in real time, which would assist in determining the combination of targeted and chemotherapies to optimize first-line therapy.

## Author Contributions

JA wrote the first draft of this article, which served as the main body of this work. JA identified and verified sources, and physically created the figure. DA proposed the concept of this paper, designed the figure, and provided multiple revisions of the various drafts. SM is an oncology-based surgeon who brought these clinical issues to the attention of our group, provided the de-identified patient records for our review, and contributed to the many revisions of this paper. Together, JA, DA, and SM devised this hypothesis to potentially improve patient management strategies in the esophageal cancer care arena.

## Conflict of Interest Statement

The authors have no other relevant affiliations or financial involvement with any organization or entity with financial interest or financial conflict with the subject matter or materials discussed in the manuscript apart from those disclosed. No writing assistance was utilized in the production of this manuscript.

## References

[1] Kailasam A Mittal SK Agrawal DK Epigenetics in the pathogenesis of esophageal adenocarcinoma. Clin Transl Sci (2015) 8:394–402. 10.1111/cts.12242 25388215 PMC4429045

[2] Howlader N Noone AM Krapcho M Garshell J Miller D Altekruse SF, editors. SEER Cancer Statistics Review, 1975–2011. Bethesda, MD: National Cancer Institute (2013). Available from: http://seer.cancer.gov/csr/1975_2011/

[3] Sehdev A Catenacci DVT. Perioperative therapy for locally advanced gastroesophageal cancer: current controversies and consensus of care. J Hematol Oncol (2013) 6:66. 10.1186/1756-8722-6-66 24010946 PMC3844370

[4] Wiedmann MW Mössner J. New and emerging combination therapies for esophageal cancer. Cancer Manag Res (2013) 5:133–46. 10.2147/CMAR.S32199 23869177 PMC3706320

[5] Iqbal N Iqbal N. Human epidermal growth factor receptor 2 (HER2) in cancers: overexpression and therapeutic implications. Mol Biol Int (2014) 2014:852748. 10.1155/2014/852748 25276427 PMC4170925

[6] Nagaraja V Eslick GD. HER2 expression in gastric and oesophageal cancer: a meta-analytic review. J Gastrointest Oncol (2015) 6:143–54. 10.3978/j.issn.2078-6891.2014.107 25830034 PMC4311097

[7] Davidson M Starling N Trastuzumab in the management of gastroesophageal cancer: patient selection and perspectives. Onco Targets Ther (2016) 9:7235–45. 10.2147/OTT.S100643 27932891 PMC5135398

[8] Onitilo AA Engel JM Stankowski RV. Cardiovascular toxicity associated with adjuvant trastuzumab therapy: prevalence, patient characteristics, and risk factors. Ther Adv Drug Saf (2014) 5:154–66. 10.1177/2042098614529603 25083270 PMC4110857

[9] Dragovich T Campen C. Anti-EGFR-targeted therapy for esophageal and gastric cancers: an evolving concept. J Oncol (2009) 2009:804108. 10.1155/2009/804108 19636422 PMC2712675

[10] Brahmer JR Tykodi SS Chow LQM Hwu W-J Topalian SL Hwu P Safety and activity of anti-PD-L1 antibody in patients with advanced cancer. N Engl J Med (2012) 366:2455–65. 10.1056/NEJMoa1200694 22658128 PMC3563263

[11] Topalian SL Drake CG Pardoll DM. Targeting the PD-1/B7-H1(PD-L1) pathway to activate anti-tumor immunity. Curr Opin Immunol (2012) 24:207–12. 10.1016/j.coi.2011.12.009 22236695 PMC3319479

[12] Rose MG Farrell MP Schmitz JC. Thymidylate synthase: a critical target for cancer chemotherapy. Clin Colorectal Cancer (2002) 1:220–9. 10.3816/CCC.2002.n.003 12450420

[13] Yin M Yan J Martinez-Balibrea E Graziano F Lenz HJ Kim HJ ERCC1 and ERCC2 polymorphisms predict clinical outcomes of oxaliplatin-based chemotherapies in gastric and colorectal cancer: a systemic review and meta-analysis. Clin Cancer Res (2011) 17:1632–40. 10.1158/1078-0432.CCR-10-2169 21278243 PMC3060288

[14] Olaussen KA Dunant A Fouret P Brambilla E André F Haddad V DNA repair by ERCC1 in non-small-cell lung cancer and cisplatin-based adjuvant chemotherapy. N Engl J Med (2006) 355:983–91. 10.1056/NEJMoa060570 16957145

[15] Bepler G Williams C Schell MJ Chen W Zheng Z Simon G Randomized international phase III trial of ERCC1 and RRM1 expression-based chemotherapy versus gemcitabine/carboplatin in advanced non-small-cell lung cancer. J Clin Oncol (2013) 31:2404–12. 10.1200/JCO.2012.46.9783 23690416 PMC3691357

[16] Arriola E Rodriguez-Pinilla SM Lambros MBK Jones RL James M Savage K Topoisomerase II alpha amplification may predict benefit from adjuvant anthracyclines in HER2 positive early breast cancer. Breast Cancer Res Treat (2007) 106:181–9. 10.1007/s10549-006-9492-5 17260090

[17] Braun MS Richman SD Quirke P Daly C Adlard JW Elliott F Predictive biomarkers of chemotherapy efficacy in colorectal cancer: results from the UK MRC FOCUS trial. J Clin Oncol (2008) 26:2690–8. 10.1200/JCO.2007.15.5580 18509181

[18] Teicher BA. Next generation topoisomerase I inhibitors: rationale and biomarker strategies. Biochem Pharmacol (2008) 75:1262–71. 10.1016/j.bcp.2007.10.016 18061144

[19] Yang Y-L Luo X-P Xian L. The prognostic role of the class III β-tubulin in non-small cell lung cancer (NSCLC) patients receiving the taxane/vinorebine-based chemotherapy: a meta-analysis. PLoS One (2014) 9:e93997. 10.1371/journal.pone.0093997 24705847 PMC3976369

[20] Karki R Mariani M Andreoli M He S Scambia G Shahabi S βIII-tubulin: biomarker of taxane resistance or drug target? Expert Opin Ther Targets (2013) 17:461–72. 10.1517/14728222.2013.766170 23379899

[21] Hou Z Orr S Matherly LH Post-transcriptional regulation of the human reduced folate carrier as a novel adaptive mechanism in response to folate excess or deficiency. Biosci Rep (2014) 34:e00130 10.1042/BSR20140065 24949876 PMC4122975

[22] De Laurentiis M Cancello G Zinno L Montagna E Malorni L Esposito A Targeting HER2 as a therapeutic strategy for breast cancer: a paradigmatic shift of drug development in oncology. Ann Oncol (2005) 16:iv7–13. 10.1093/annonc/mdi901 15923434

[23] Rybak LP Mukherjea D Jajoo S Ramkumar V. Cisplatin ototoxicity and protection: clinical and experimental studies. Tohoku J Exp Med (2009) 219:177–86. 10.1620/tjem.219.177 19851045 PMC2927105

[24] Larkin J Chiarion-Sileni V Gonzalez R Grob JJ Cowey CL Lao CD Combined nivolumab and ipilimumab or monotherapy in untreated melanoma. N Engl J Med (2015) 373:23–34. 10.1056/NEJMoa1504030 26027431 PMC5698905

[25] Raufi AG Klempner SJ. Immunotherapy for advanced gastric and esophageal cancer: preclinical rationale and ongoing clinical investigations. J Gastrointest Oncol (2015) 6:561–9. 10.3978/j.issn.2078-6891.2015.037 26487950 PMC4570917

[26] Sparano JA Gray RJ Makower DF Pritchard KI Albain KS Hayes DF Prospective validation of a 21-gene expression assay in breast cancer. N Engl J Med (2015) 373:2005–14. 10.1056/NEJMoa1510764 26412349 PMC4701034

[27] Nuciforo P Thyparambil S Aura C Garrido-Castro A Vilaro M Peg V High HER2 protein levels correlate with increased survival in breast cancer patients treated with anti-HER2 therapy. Mol Oncol (2016) 10:138–47. 10.1016/j.molonc.2015.09.002 26422389 PMC4968773

